# First corpus callosotomy for medically refractory epilepsy in The Gambia: an international cooperation case report and historical review

**DOI:** 10.1186/s42494-026-00258-2

**Published:** 2026-06-01

**Authors:** Zsombor T. Gal, Ebrima K. Manneh, Saksham Gupta, Gabrielle A. Luiselli, Makumba Cham, Ancha Ceesay, Mai Nyassi, Yusupha Jobe, Richard Oguocha, Musa Barry, Mary F. Gomez, Malick Jammeh, Oley Bojang, Tida Jagne, Fatoumata Jallow, Adama Njie, Cherno S. Jallow, Mustapha Bittaye, Mariam Joof, Kebba S. Marenah, Yohana C. Sanchez, Maguette Mbaye, Nantenin Doumbia, Sabina Kangakan, Pokua Sarpong, Mhd A. Alkhateeb, Eduardo R. Cobas, Lamin Janneh, John D. Rolston, John N. Jabang

**Affiliations:** 1 Department of Neurosurgery, Mass General Brigham, Harvard Medical School, Boston, MA 02115 USA; 2 Neurosurgery Unit of the Department of Surgery of Edward Francis Small Teaching Hospital, University of The Gambia, Banjul, 00000 The Gambia; 3 Service de Neurochirurgie, Centre Hospitalier National Universitaire de Fann, BP 5035 Dakar, Senegal; 4 Department of Anesthesia, Edward Francis Small Teaching Hospital, University of The Gambia, Banjul, 00000 The Gambia

**Keywords:** Epilepsy surgery, Corpus callosotomy, Drop attacks, The Gambia, Global neurosurgery

## Abstract

**Background:**

Epilepsy affects over 50 million people worldwide, with the majority residing in low- and middle-income countries (LMICs) where access to specialized care is often constrained. Surgical intervention represents a crucial therapeutic modality for patients with medically refractory epilepsy, yet epilepsy surgery remains markedly underutilized in sub-Saharan Africa.

**Case presentation:**

We report the case of a 10-year-old boy in The Gambia with medically refractory epilepsy characterized by daily drop attacks. Despite optimized and adequately trialed therapy with carbamazepine and sodium valproate, he continued to experience disabling seizures, leading to recurrent head trauma. Computed tomography (CT) and magnetic resonance imaging (MRI) did not reveal an identifiable structural lesion. Given the intractability of seizures and significant associated morbidity, he underwent a corpus callosotomy. The procedure was performed via a standard microsurgical approach and concurrently addressed the resection of traumatic calcified scalp hematomas. The patient’s postoperative recovery was uneventful, and he was discharged on postoperative day 13 and remained seizure-free. At the six-month postoperative follow-up, the patient achieved complete freedom from drop attacks, with generalized tonic–clonic seizures (GTCS) occurring only during periods of antiseizure medication (ASM) interruption.

**Conclusions:**

This case highlights the feasibility and therapeutic potential of epilepsy surgery in the context of a surgical program within a resource-limited setting. Scaling up access to epilepsy surgery in comparable environments could contribute to mitigating the global epilepsy treatment gap.

## Background

Epilepsy is a chronic neurological disorder characterized by recurrent seizures, affecting over 50 million people globally, with nearly 80% residing in low- and middle-income countries (LMICs) [1]. In these settings, the epilepsy treatment gap may exceed 75% [2]. Sub-Saharan Africa bears a particularly high burden of epilepsy, driven by factors such as infectious etiologies, birth trauma, genetic predisposition, and constrained access to healthcare [3, 4]. Antiseizure medications (ASMs) constitute the standard first-line treatment; however, the African region reports the lowest mean utilization of ASMs, at approximately 24.5%, which reflects inadequate access to pharmacotherapy and contributes significantly to the substantial epilepsy treatment gap [5]. Moreover, studies from high-income countries indicate that up to one-third of individuals with epilepsy develop drug-resistant disease [6]. In contrast, the epidemiology and determinants of treatment refractoriness in LMICs remain less clearly delineated and may differ due to variations in etiology, treatment accessibility, and health system factors. For patients with medically refractory epilepsy, surgical interventions may substantially reduce seizure burden, thereby improving quality of life.

Globally, it is estimated that more than 10 million patients with epilepsy may benefit from surgical intervention, with the greatest proportion of potential surgical candidates in LMICs, particularly in Africa and Latin America [5]. The high burden of surgical candidates in LMICs largely primarily represents a backlog of unmet need rather than a higher incidence of epilepsy, as limited availability of epilepsy surgery services results in the accumulation of patients who are medically refractory and surgically eligible. A recent review highlighted that publications on epilepsy surgery originated from only 22% of LMICs, reflecting the profound disparity in access to epilepsy surgery [7].

While certain epilepsy surgical modalities, such as responsive neurostimulation and vagus nerve stimulation, pose significant implementation challenges in LMICs due to their dependence on expensive implants, long-term device management, and specialized follow-up, other surgical strategies—including lesionectomy or focal cortical resection for well-defined structural lesions—can be effectively pursued with available resources [8]. For patients in whom a resectable epileptogenic focus cannot be identified, palliative disconnection surgeries like corpus callosotomy (CC) represent a valuable therapeutic alternative, especially for those suffering from disabling drop attacks [9]. The presurgical evaluation for potential CC candidates typically initiates with structural neuroimaging. In many low-resource settings, this evaluation relies on computed tomography (CT) and conventional magnetic resonance imaging (MRI), which may not detect subtle cortical abnormalities. Conversely, high-income settings routinely utilize epilepsy-protocol high-resolution MRI and adjunctive functional imaging modalities like positron emission tomography or magnetoencephalography, which are frequently inaccessible in resource-limited environments. Consequently, the absence of a lesion on available imaging in these settings reflects the limitations of the diagnostic technology available rather than a definitive absence of focal pathology. Large clinical series from high-volume epilepsy centers have demonstrated sustained reductions in drop attacks and acceptable neurocognitive profiles following CC, including evidence from institutions like the Montreal Neurological Institute and other tertiary epilepsy centers [10–12]. Importantly, CC has been successfully adopted in LMICs, where it has yielded significant reductions in seizure burden and associated injuries, even within substantial infrastructural constraints [13–15].

In The Gambia, a West African nation facing considerable healthcare resource constraints, the capacity for neurosurgical care has been historically underdeveloped [16, 17]. Until recently, patients requiring surgical treatment for epilepsy often had no access to definitive intervention, managing their condition solely with pharmacological therapy of variable efficacy. Here, we present a case of CC performed in The Gambia as part of an emerging epilepsy surgery program, focusing on the feasibility, the multidisciplinary coordination, and the perioperative considerations necessary in a resource-limited setting. This case aims to share insights and specific practice with global epilepsy community on initiating surgical epilepsy care in environments where such interventions are newly being established.

## Case presentation

The patient is a 10-year-old boy with medically refractory epilepsy characterized predominantly by frequent drop attacks, occurring up to ten times daily, alongside generalized tonic–clonic seizures (GTCS) occurring up to three times daily. No focal motor seizures were reported. Assessment of auras was unreliable due to the patient’s young age and baseline expressive language impairment, and no consistent pre-ictal behaviors were observed by caregivers. Seizures were first noted at 10 months of age. Prior to surgical referral, he had been treated with carbamazepine and subsequently sodium valproate, as these were the primary ASMs accessible to the family. No other ASMs were tried due to limited access and financial constraints. He tolerated both agents without documented adverse effects; however, neither adequately control his drop attacks, which remained frequent and disabling.

**Fig. 1:**
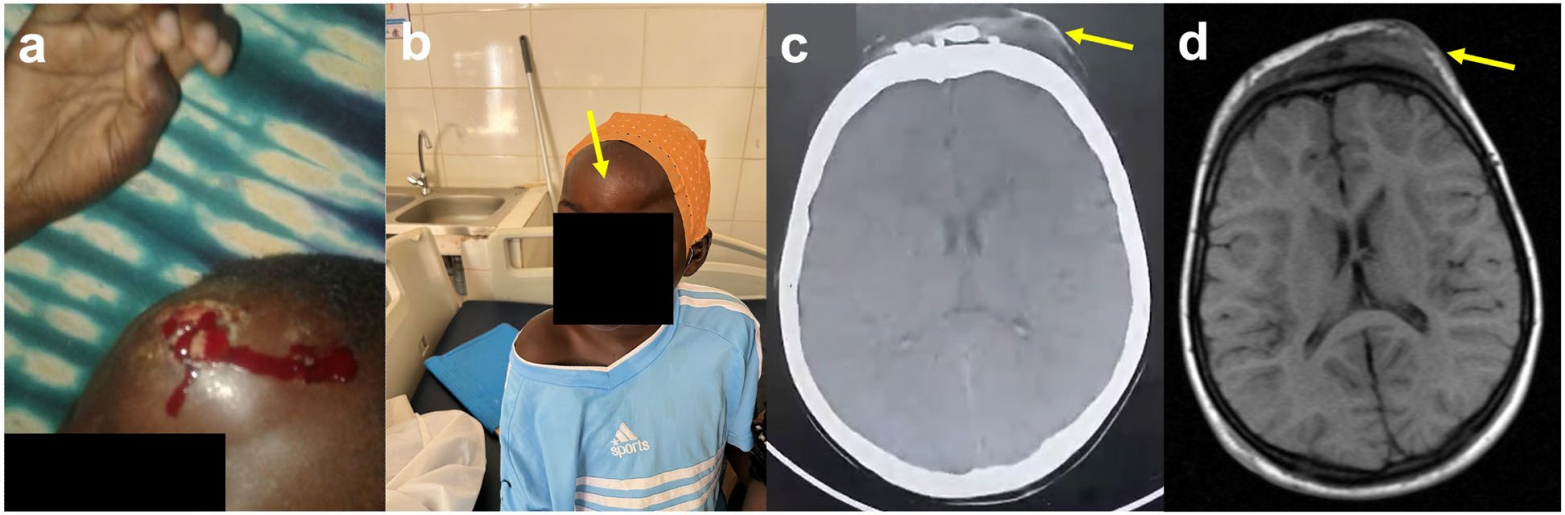
Preoperative findings. **a** Traumatic forehead injury sustained during drop attacks. **b** Forehead chronic traumatic scalp lesions resulted from repeated head impacts during drop attacks (yellow arrow). **c** CT demonstrated prominent forehead callosities (yellow arrow) without underlying structural lesion. **d** MRI showed forehead lesions (yellow arrow) but otherwise confirming the absence of focal pathology

On presentation to the neurosurgery clinic at Edward Francis Small Teaching Hospital, the patient was nonverbal but maintained eye contact, smiled, interacted physically with his mother, moved purposefully, and ambulated independently. Examination revealed multiple chronic traumatic forehead lesions and calcified subgaleal hematomas, attributable to repeated head impacts from longstanding drop attacks (Fig. 1a-b).

Developmentally, the patient had significant delays in expressive language and had received no formal education. He was nonverbal at baseline and had not attended school prior to surgery, owing mainly to the frequency of his seizures, safety concerns from drop attacks, and limited access to specialized educational resources. Despite this, he demonstrated social engagement with caregivers, purposeful movement, and independent ambulation. The patient required constant supervision due to the frequency and unpredictability of his drop attacks, imposing a substantial caregiving burden on his family. His mother served as the primary caregiver and reported difficulty managing activities of daily living outside the home because of safety concerns regarding sudden falls and head injuries. These caregiving demands, along with limited access to protective equipment and specialized support services, significantly impacted the family’s daily functioning.

Cranial CT (Figs. 1c) and MRI (1d) confirmed calcified frontal scalp lesions and an intact corpus callosum, with no evidence of intracranial structural abnormalities. Preoperative scalp electroencephalography (EEG), interpreted in collaboration with an international tele-EEG service, demonstrated generalized epileptiform discharges without a consistent focal onset or lateralizing features. These findings, in conjunction with non-lesional neuroimaging and the predominance of injurious drop attacks, supported the decision to pursue a palliative disconnection procedure.

Regarding feasibility, this intervention was enabled by a combination of existing local surgical capacity and international collaboration. The procedure was performed using standard microsurgical instruments, an operating microscope, and routinely available anesthesia equipment, with perioperative care provided by the local multidisciplinary team. Specialized resources such as long-term video-EEG monitoring, intracranial electroencephalography (iEEG) monitoring, and formal neuropsychological assessment were not available; therefore, preoperative evaluation was based on clinical semiology, available scalp EEG, and structural neuroimaging. International epilepsy surgery collaborators provided consultative support and surgical mentorship, facilitating safe patient selection and operative planning within the existing resource constraints.

The patient’s case was discussed in a multidisciplinary discussion involving local neurosurgery and neurology teams, anesthesia providers, and international epilepsy surgery collaborators. Given the predominance of frequent, injurious drop attacks, non-lesional neuroimaging, and generalized epileptiform activity without lateralizing features on EEG, a total CC was chosen to maximize the likelihood of reducing atonic seizures. This decision was informed by prior evidence demonstrating superior control of drop attacks with complete callosal sectioning compared with anterior callosotomy alone [18]. Informed consent was obtained from the patient’s parent.

**Fig. 2:**
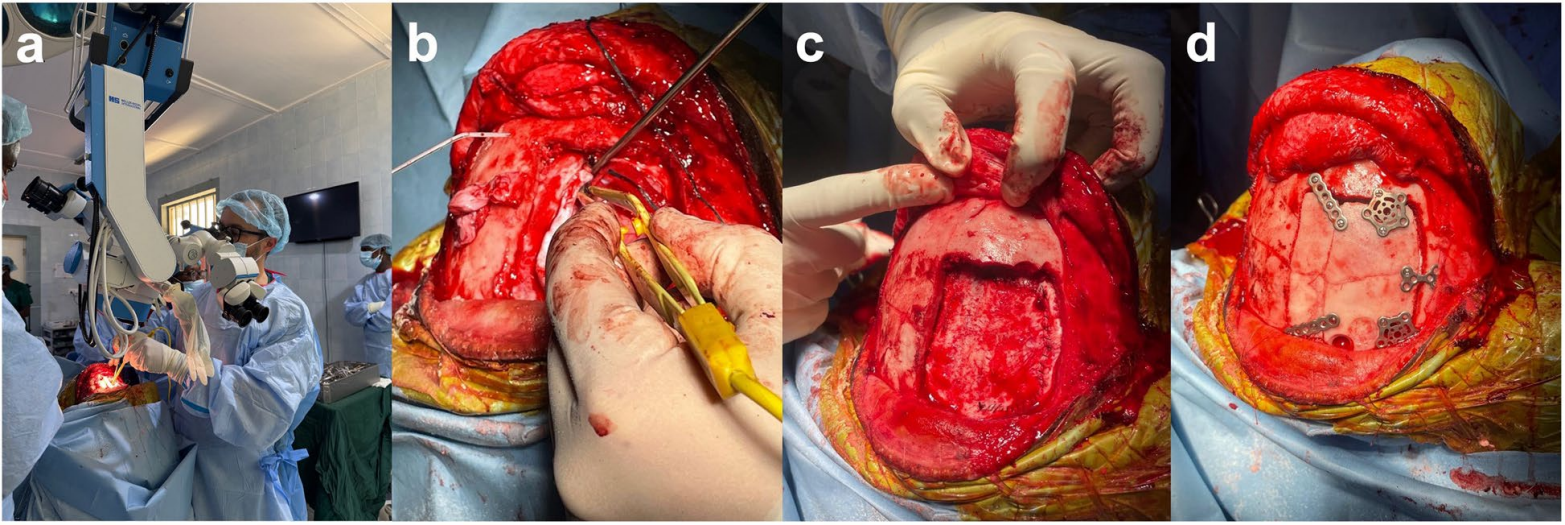
Intraoperative images. **a** Surgical microscope employed for the corpus callosotomy. **b** Bipolar cautery and rolled surgical patties used to complete the interhemispheric approach. **c** Dural closure completed with non-absorbable sutures. **d** Skull flap reaffixed with titanium plates and screws

The patient was positioned supine with the head slightly flexed. A coronal incision was made, followed by bifrontal craniotomy. The surgical microscope was employed (Fig. 2a). An interhemispheric approach was undertaken with retraction aided by rolled surgical patties (Fig. 2b). Under magnification, the corpus callosum was identified and sectioned in a posterior trajectory from rostrum to splenium, using the Vein of Galen as a posterior landmark. The dura was closed with non-absorbable sutures (Fig. 2c), and the bone flap was secured with titanium plates and screws (Fig. 2d). Following the CC, the frontal scalp hematomas were burred down flush with the adjacent calvarium. The scalp was closed in layers with absorbable and non-absorbable sutures. The patient was extubated immediately postoperatively and transferred to the recovery ward.

The postoperative course was uncomplicated. On postoperative day one (POD1), the patient was drowsy and kept *nil per os.* Oral intake was resumed on POD2 following cognitive improvement. Physiotherapy and ambulation were initiated on POD4. No postoperative seizures were reported during the inpatient hospitalization. Perioperatively, he received intravenous ceftriaxone, which was later switched to oral amoxicillin-clavulanate, and was maintained on intravenous phenytoin alongside standard analgesics (paracetamol, tramadol, and diclofenac). The family was advised to continue his preoperative ASM regimen for at least six months, with a view to gradually tapering the medications if he remained seizure-free. The patient was discharged home at his neurologic baseline on POD13.

At six-month follow-up, the patient’s mother reported complete cessation of drop attacks since surgery. GTCS recurred only during episodes of ASM non-adherence due to financial difficulties and were otherwise controlled with consistent dosing. No new seizure types were reported. Functionally, the patient returned to his neurologic baseline postoperatively. Caregivers reported improved safety, less need for constant supervision, and fewer traumatic injuries. While formal neuropsychological testing was not available, there was no observed postoperative cognitive decline or behavioral deterioration. No surgical complications occurred, intra- or postoperatively, such as infection, hemorrhage, or new neurologic deficits. Postoperative MRI was not obtained due to the prohibitive out-of-pocket cost.

## Discussion

This case illustrates the feasibility of implementing CC as part of an emerging epilepsy surgery program in a resource-limited setting like The Gambia. While the surgical technique and seizure outcomes of CC are well-established, the value of this report lies in its implementation-focused perspective. It highlights the multidisciplinary coordination, logistical considerations, and system-level challenges inherent to delivering epilepsy surgery where such care was previously unavailable.

Several epilepsy surgeries can be feasible in resource-limited settings, particularly those requiring minimal technological and infrastructural investment. Highly effective surgical procedures such as CC, lesionectomy for well-defined focal lesions, hemispherectomy for hemispheric syndromes, and anterior temporal lobectomy can often be performed successfully with relatively limited surgical and diagnostic facilities. In contrast, procedures like iEEG monitoring or laser interstitial thermal therapy (LITT) demand substantial resources and specialized expertise, typically limiting their availability in low-resource contexts [7].

### Historical context

The introduction of epilepsy surgery in The Gambia marks a milestone in the advancement of neurosurgical care within the country. Historically, epilepsy care in The Gambia has been hindered by widespread stigma, insufficient healthcare infrastructure, and scarcity of neurologists and neurosurgeons [19]. Early reports from The Gambia documented a lifetime epilepsy prevalence of 4.9 per 1000. Fewer than one in ten patients could maintain continuous treatment, despite 80% being potentially controllable with low-cost ASMs [19, 20]. At the time, only phenytoin, phenobarbital, and carbamazepine were widely available. Notably, all surveyed patients had sought traditional remedies—such as exorcisms, amulets, and herbal treatments—reflecting prevailing beliefs in spiritual causes of epilepsy [19, 20]. Stigma was profound, with many individuals reportedly disowned or excluded from school and employment. Nevertheless, 61% indicated a preference for preventive biomedical treatment if it were locally accessible [19, 20]. These findings underscore how cultural beliefs, poverty, and limited infrastructure—including the absence of EEG capacity, only one neurologist, and no neurosurgeons [20]—combined to perpetuate the treatment gap. Collaboration between local medical teams and international partners has been instrumental in addressing these barriers, helping to build a neurosurgical workforce, train providers in epilepsy management, and laying the groundwork for introducing epilepsy surgery in The Gambia [17, 20].

### Feasibility and scalability

This case confirms that CC is technically feasible in a low-resource setting when basic neurosurgical infrastructure, anesthesia support, and postoperative care are available. A key advantage is that the procedure does not require advanced implants or specialized consumables, making it more adaptable for implementation than other epilepsy surgery modalities. Preoperative EEG remains a fundamental component of epilepsy surgery evaluation and should be obtained whenever possible to characterize seizure type and guide surgical planning. Expanding access to both routine and long-term EEG monitoring remains a critical priority for developing epilepsy surgery programs in low- and middle-income countries.

From a family perspective, the pathway to surgery involved repeated healthcare visits, time away from work and home responsibilities for caregivers, and challenges with medication adherence, often disrupted by financial constraints. Travel to the surgical center and the intensive caregiving demands necessitated by frequent, injurious drop attacks further added to the family burden. Although this report did not systematically quantify the direct and indirect costs of care, it illustrates how financial and logistical constraints can significantly influence both preoperative management and postoperative seizure control in low-resource environments.

However, broader availability of this intervention in the region remains constrained by workforce shortages, inconsistent access to ASMs, and the lack of comprehensive epilepsy monitoring infrastructure. Currently, such interventions are most viable within structured programs supported by sustained local training and international collaboration, rather than as a broadly available standard-of-care. Expansion to more patients will require continued investment in neurosurgical capacity, epilepsy care pathways, and longitudinal follow-up systems.

### The role and future of epilepsy surgery in The Gambia

In LMICs, the effectiveness of ASMs depends not only on appropriate drug selection and dosing, but also on uninterrupted, long-term access. Patients and families face recurrent barriers to continuous medication use, including financial constraints, inconsistent drug supply, long distance to pharmacies or hospitals, and health system disruptions. Even brief interruptions in ASM therapy can precipitate seizure recurrence and injury, as observed in this case. These realities complicate the management of drug-resistant epilepsy in resource-limited settings and contribute to persistent seizure burden despite otherwise appropriate medical therapy. In this context, surgical interventions like CC may play a complementary role by reducing the frequency of the most injurious seizure types and lessening the reliance on perfectly sustained medication access alone, though they do not eliminate the need for continued antiseizure therapy.

Expanding epilepsy surgery in The Gambia now holds the potential for substantial medical and economic benefits. By reducing seizure-related injuries, improving quality of life, and decreasing long-term dependence on pharmacotherapy and hospitalizations, surgery offers a durable solution where medication alone is often insufficient. In children, earlier surgical intervention may help prevent cumulative neurocognitive decline, enabling better educational outcomes and social integration. Beyond seizure reduction, epilepsy surgery in this context has important implications for family well-being. Frequent drop attacks required continuous supervision, limiting caregiver mobility, employment opportunities, and participation in daily activities. Reducing seizure burden, therefore, has the potential to alleviate not only patient morbidity but also substantial caregiving and social strain on families in low-resource settings. Despite these advantages, epilepsy surgery remains underperformed in The Gambia and other LMICs due to multifactorial barriers: a limited number of trained epileptologists, lack of advanced diagnostics such as continuous EEG monitoring, persistent social stigma and cultural misconceptions about epilepsy, and ongoing health systems constraints [19, 21]. In parallel, community-based organizations like the Foundation for Epilepsy and Stigma Support in The Gambia (FESSGAM) have intensified nationwide campaigns to dispel misconceptions, reduce stigma, and promote biomedical treatment. These efforts complement hospital-based progress and encourage earlier health-seeking behaviors. Through radio, television, and social media, FESSGAM has engaged communities in dialogue about epilepsy. Their outreach also provides practical family support and advocate for policy changes, positioning the organization as a critical partner in expanding access to care. In collaboration with TeleEEG in the United Kingdom, FESSGAM has ensured the availability of EEG services in The Gambia since August 2021.

This case underscores the critical role for surgical options in managing epilepsy in LMICs, where pharmacologic treatment alone is frequently inadequate. Building sustainable epilepsy surgery programs will require the continued development of local expertise, strengthened by international collaborations, to ensure program viability, broader global access to epilepsy surgery, and improved outcomes for affected populations [21]. Recent reports highlight both progress in neurosurgical service delivery and persistent bottlenecks in infrastructure and workforce, situating this case within a broader movement to expand epilepsy care in The Gambia [16]. Future initiatives in The Gambia and similar contexts should prioritize expanding training opportunities, enhancing diagnostic capacity, and fostering community awareness to mitigate stigma and improve patient outcomes. Finally, emerging digital tools, such as large language models and generative artificial intelligence, may aid in surgical case identification and patient triage for epilepsy surgery at the Edward Francis Small Teaching Hospital and similar centers.

### Limitations and future directions

This report is limited by its single-patient design, relatively short follow-up duration, and the absence of standardized quality-of-life assessments. Follow-up in this report is limited to six months postoperatively, and longer-term seizure outcomes, including one-year follow-up, will be important to assess the durability of surgical benefit as the program matures. Assessment of neurocognitive adverse effects, such as disconnection syndrome, was limited by the lack of formal neuropsychological testing, and relied on clinical observation and caregiver report during short-term follow-up.

While longer-term seizure outcomes and patient-reported measures are essential, systematic longitudinal data collection is still under development within the emerging epilepsy surgery program in The Gambia. Ongoing efforts within this international collaboration are focused on prospective patient enrollment, standardized seizure outcome reporting, and incorporating quality-of-life metrics as program capacity and follow-up infrastructure mature. Future efforts within this collaboration aim to establish prospective case series and, where feasible, a local epilepsy registry to enable systematic outcome tracking and guide program development.

## Conclusions

This case illustrates the feasibility of CC within a developing epilepsy surgery program in a low-resource setting. The patient achieved resolution of drop attacks post-surgery. The overall postoperative seizure outcome corresponding to Engel Class ID, due to the persistence of GTCS during periods of ASM interruption. Beyond the individual outcome, this experience represents a broader milestone in the development of neurosurgical capacity in The Gambia, where epilepsy remains a significant public health challenge. Expanding access to epilepsy surgery through local training, international collaboration, and targeted infrastructure investment will be essential to narrowing the treatment gap and reducing the burden of uncontrolled seizures in similar contexts.

## Data Availability

All data generated or analyzed during this study are included in this published article.

## Abbreviations

ASMs: Antiseizure medications
CC: Corpus callosotomy
CT: Computed tomography
EEG: Electroencephalography
FESSGAM: Foundation for Epilepsy and Stigma Support in The Gambia
GTCS: Generalized tonic–clonic seizures
iEEG: Intracranial electroencephalography
LITT: Laser interstitial thermal therapy
LMICs: Low- and middle-income countries
MRI: Magnetic resonance imaging
POD: Postoperative day
